# MALDI-TOF MS based carbapenemase detection from culture isolates and from positive blood culture vials

**DOI:** 10.1186/s12941-016-0120-x

**Published:** 2016-02-02

**Authors:** B. Ghebremedhin, A. Halstenbach, M. Smiljanic, M. Kaase, P. Ahmad-Nejad

**Affiliations:** Institute for Medical Laboratory Diagnostics, Center for Clinical and Translational Research, Witten/Herdecke University, Heusnerstr. 40, 42283 Wuppertal, Germany; Department of Medical Microbiology, Ruhr-University Bochum, Bochum, Germany

**Keywords:** MALDI-TOF MS, Carbapenemases, *Acinetobacter baumannii*, MDR Gram-negative bacteria, Hydrolysis

## Abstract

**Background:**

Antibiotic resistance in bacteria leads to massive health problems. Incidence of carbapenem and multidrug resistance in Gram-negative bacteria are increasing globally and turn out to be a very urgent challenge in health care. Resistant bacteria play an important clinical role during hospital outbreaks as well as in sepsis. Rapid diagnostic tests are necessary to provide immediate information for antimicrobial treatment and infection control measures.

**Methods:**

Our mass spectrometry-based assay was validated with 63 carbapenemase-producing Gram-negative bacterial isolates, and 35 carbapenem-resistant Gram-negative species with no carbapenemase production. These were analyzed from solid culture media and positive blood culture vials. After 4 h of incubation the carbapenemase products were analyzed with the MALDI-TOF MS. All the isolates were genotyped for carbapenemase genes by PCR and sequencing.

**Results:**

For culture isolates the concordance of hydrolysis assay to genetic results was 98 % for OXA variants, KPC, VIM, IMP, GIM, and NDM. In contrast, only 14 of 29 *Acinetobacter baumannii* isolates carrying the OXA and NDM genes could be identified from blood culture. However, from blood culture vials our method allowed the detection of carbapenemases in 98 % of *Pseudomonas* and *Enterobacteriaceae* isolates harboring different genes.

**Conclusions:**

This MALDI-TOF MS–based assay permitted the detection of carbapenemases either from solid culture media (98–100 %) or blood culture vials (96 %) for all non-*A. baumannii* isolates within 4 h. In case of *A. baumannii* isolates the assay was highly sensitive for the detection of carbapenemases directly from solid culture media.

## Background

The resistance of bacteria to antibiotics has increased in recent decades. Resistant bacteria can significantly complicate the treatment of infections in critically ill patients; especially in surgery, hemato-oncology, and intensive care in general [1, 2]. Recently, bacterial isolates that are resistant to all available antibiotics have also been emerging [3, 4]. The identification and detection of specific resistance mechanisms in these bacteria are of paramount importance to clinical microbiologists for diagnosis and treatment of systemic infections, such as blood stream infections (BSIs), which allows for effective management of systemic infections, which allows de-escalation from broad spectrum to targeted antibiotics, thus reducing overuse of carbapenems and other broad spectrum antibiotics. The diagnostic methods for detection of carbapenemase producers include at first level the antibiotic susceptibility testing results obtained by disk diffusion or automated systems, E-test and the verification via modified Hodge test. However, the later method may lack specificity (high-level AmpC producers) and sensitivity, e.g. weak detection of NDM variants. Several technologies using molecular methods have been developed in recent years (i.e., targeted PCR assays [5, 6] and peptide nucleic acid fluorescent in situ hybridization [7, 8]). Nowadays, Matrix-assisted laser desorption ionization–time of flight mass spectroscopy (MALDI-TOF MS) is increasingly utilized by clinical microbiology laboratories for the identification of bacteria and yeasts [9, 10]. MALDI-TOF MS has been performed for the detection of antimicrobial resistance, e.g. β-lactam resistance in Gram-negative bacteria [11, 12]. For instance, this method is effective for detection of carbapenemase-producing gram-negative bacteria [9, 12–15].

## Methods

### Bacterial isolates

Sixty three Gram-negative bacteria, comprised of *Enterobacteriaceae* and non-fermenters, were collected by the National Reference Center for Gram-negative bacteria at the Ruhr University Bochum and at the Institute in Wuppertal. All of the isolates had been previously characterized at the molecular level for the diverse types of carbapenemases (KPC, GIM, IMP, NDM, OXA, and VIM). As negative control, 35 non-carbapenemase-producing, but carbapenem-resistant isolates were included. Species identification was performed using a MALDI-TOF Biotyper system (Bruker Daltonics, Bremen, Germany) and phenotypic antimicrobial susceptibility testing by use of Phoenix™ automated system (Becton–Dickinson, Heidelberg, Germany), including ampicillin (± sulbactam), piperacillin (± tazobactam), cefuroxime, cefotaxime, cefepime, ceftazidime, imipenem, meropenem, gentamicin, ciprofloxacin, levoflocaxin, co-trimoxazole, amikacin, fosfomycin, tigecycline, and colistin.

### Imipenem hydrolysis assay

A colony of an overnight bacterial culture on MacConkey agar (Becton–Dickinson, Heidelberg, Germany) was washed in 1 ml H_2_O dest. and centrifuged at 13,200 rpm for 1 min, the harvested pellet was resuspended in 20 mM Tris–HCl, 20 mM NaCl, pH 7.0, to an inoculum equivalent to 3.0 McFarland standard. A 1-ml aliquot of the suspension was centrifuged at 13,200 rpm for 1 min; the harvested pellet was resuspended in 50 µl of a reaction buffer (20 mM Tris–HCl), pH 7.0, supplemented with 0.1 mM imipenem (Fresenius Kabi, Bad Homburg, Germany). Four hours after incubation at 37 °C in an Eppendorf thermomixer [agitation of 950 rpm] (Eppendorf, Hamburg, Germany) the reaction mixture was centrifuged at 13,200 rpm for 1 min; 1 µl of the supernatant was mixed with 1 µl HCCA and allowed to dry on a target. Mass spectra were measured after drying between 160 and 700 *m/z*, using Microflex LT mass spectrometer. Spectra were analyzed using the software program FlexControl, version 3.0 (Bruker Daltonics, Bremen, Germany). For the analysis of the hydrolysis activity from blood culture vials containing the Gram-negative bacterial isolates, 8 ml of blood culture—after 10 min at 800 rpm—the supernatant was mixed with 200 μl of lysis buffer. After vortexing, the sample was centrifuged at 13,200 rpm for 2 min, the supernatant was discarded, and the pellet was washed with 1 ml of deionized H_2_O. The water was thoroughly removed, and the bacterial pellets (10^7^–10^9^ CFU) were resuspended in 10 μl of 0.1 mM imipenem solution and SDS (2 %). The suspension was incubated at 37 °C as mentioned above. Thereafter centrifugation at 13,200 rpm for 2 min followed and the cell-free supernatant was analyzed by MALDI-TOF–MS.

The absence of the imipenem + matrix component peak at 479 *m/z* (299 + 180 *m/z*) was considered as positive for the carbapenemase production, indicating the peaks of the degradation products with overlap of mass peaks. For non-carabapenemase-producing isolates, the complex peak of imipenem/matrix was recorded at 479 *m/z*. According to these findings the interpretation criteria were established.

## Results

### Identification of relevant peaks in the MS-spectra after hydrolysis assay

To generate and optimize our hydrolysis assay, we performed MALDI-TOF MS analysis following different periods of incubation with imipenem (1, 2, 3, and 4 h—data not shown). The assay was conducted with 3.0 of McFarland bacterial inoculum and 0.1 mM imipenem. Although after 2 h incubation time the KPC activity was measurable, for other enzyme variants 4 h of incubation was required to demonstrate significant signals of hydrolysis in MALDI-TOF MS.

**Fig. 1:**
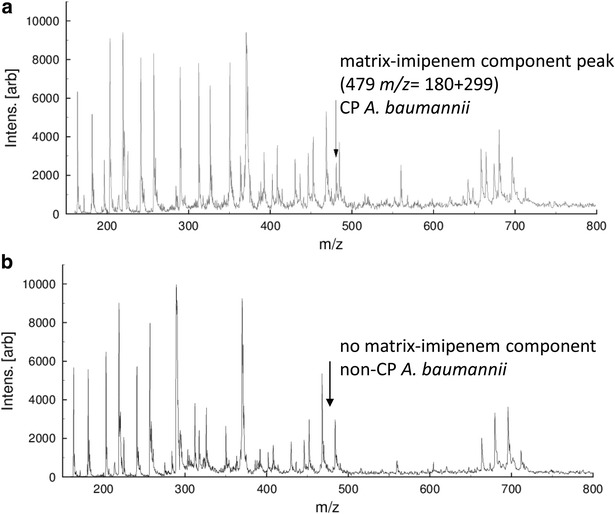
MALDI-TOF MS analysis showing the selected spectrum (range 160–800 *m/z*) after the performance of imipenem hydrolysis; OXA-23 carbapenemase-producing *A. baumannii* (**a**) and carbapenem-susceptible *A. baumannii* (**b**)

As a negative control MALDI-TOF MS analysis was performed prior to the hydrolysis assay. Differences of peaks in the spectra generated were recorded and subsequently compared to the results gained after incubation. The MS signal of imipenem-matrix complex (Fig. 1) disappeared if the bacterial isolate produced carbapenemase. For non-carbapenemase-producing species we observed the signal of the complex at 479 *m/z* (Fig. 1).

### Hydrolysis assay for carbapenemase-producing *Acinetobacter baumannii, Pseudomonas aeruginosa* and different *Enterobacteriaceae* isolates directly from solid culture media

All investigated isolates were imipenem resistant as determined by automated susceptibility testing systems (BD Phoenix). In our study 38 *A. baumannii* isolates were investigated; all were positive for the genetic presence of carbapenemases. Twenty nine of these isolates carried the OXA gene variants OXA-23, -40, -58, -72, and OXA-164; the remaining isolates were positive for NDM-1, GIM-1 and VIP-2 genes. 25 *P. aeruginosa* and *Enterobacteriaceae* isolates were positive for different carbapenemase types. Seven of the later isolates produced the enzyme variant of OXA (Table 1). The IMP variants IMP-4 and IMP-14 were detected in single *Enterobacter cloacae* isolate whereas, the NDM variants, NDM-1 or NDM-6, were carried by *Escherichia coli*, *Klebsiella pneumoniae* and *E. cloacae* isolates. The OXA variants were detected in 6 different species, but one *E. coli* isolate with OXA-48 gene did not reveal significant hydrolysis of imipenem in the MALDI-TOF MS analysis. None of the control isolates (*n* = 35, *Enterobacteriaceae* and non-fermenters), which were resistant to imipenem and meropenem but negative for the presence of a carbapenemase-encoding genes, showed hydrolysis activity in the mass spectrometry, revealing 100 % specificity for the non-carbapenemase-producing bacteria. In brief, the sensitivity of the MALDI-TOF MS-based hydrolysis assay for *A. baumannii* isolates from solid culture was 97.4 % (*n* = 37 of 38), whereas the sensitivity of 100 % was reached for the different *Enterobacteriaceae* and *P. aeruginosa* isolates. All genotyping data for other enzyme variants of carbapenemases are detailed in Table 1.

**Table 1:** Comparison of the genetic characteristics of the carbapenemase genes in *A. baumannii, Pseudomonas aeruginosa* and *Enterobacteriaceae* isolates and the activity of the respective enzymes and detection of imipenem hydrolysis by use of MALDI-TOF-MS directly from solid culture media and directly from positive blood culture vials

Bacterial isolates	Enzyme variants Ambler classification	Carbapenemase variants—genotyped	MALDI-TOF MS-based imipenem hydrolysis assay
Solid culture media			
*A. baumannii*	B (*n *= 9)	NDM-1^a^ (*n* = 7)	(*n* = 7)
		GIM-1 (*n* = 1)	(*n* = 1)
		VIM-2 (*n* = 1)	(*n* = 1)
*A. baumannii*	D (*n* = 29)	OXA-23 (*n* = 19)	(*n* = 19)
		OXA-40 (*n* = 1)	(*n* = 1)
		OXA-58 (*n* = 3)	(*n* = 3)
		OXA-72 (*n* = 4)	(*n* = 3)
		OXA-164 (*n* = 2)	(*n* = 2)
*E. coli*	A (*n* = 2)	KPC-2 (*n* = 2)	(*n* = 2)
*K. pneumoniae*	A (*n* = 3)	KPC-3 (*n* = 3)	(*n* = 3)
*E. cloacae*	B (*n* = 2)	IMP-4, IMP-14 (*n* = 2)	(*n* = 2)
*S. marcescens*	B (*n* = 1)	IMP-13	(*n* = 1)
*E. cloacae*	B (*n* = 1)	NDM-1^a^ (*n* = 1)	(*n* = 1)
*E. coli*	B (*n* = 2)	NDM-1^a^, -3 (*n* = 2)	(*n* = 2)
*K. pneumoniae*	B (*n* = 1)	NDM-1^a^ (*n* = 1)	(*n* = 1)
*K. pneumoniae*	B (*n* = 2)	VIM-2 (*n* = 2)	(*n* = 2)
*K. oxytoca*	B (*n* = 1)	VIM-1, (*n* = 1)	(*n* = 1)
*E. cloacae*	B (*n* = 1)	VIM-4 (*n* = 1)	(*n* = 1)
*P. aeruginosa*	B (*n* = 2)	VIM-2 (*n* = 2)	(*n* = 2)
*K. pneumoniae*	D (*n* = 4)	OXA-48 (*n* = 4)	(*n* = 4)
*C. freundii*	D (*n* = 1)	OXA-162 (*n* = 1)	(*n* = 1)
*E. coli*	D (*n* = 1)	OXA-181 (*n* = 1)	(*n* = 1)
*K. pneumoniae*	D (*n* = 1)	OXA-204 (*n* = 1)	(*n* = 1)
Blood culture vials			
*A. baumannii*	B (*n* = 9)	NDM-1^a^ (*n* = 7)	(*n* = 2)
		GIM-1 (*n* = 1)	(*n* = 1)
		VIM-2 (*n* = 1)	None
*A. baumannii*	D (*n* = 29)	OXA-23 (*n* = 19)	(*n* = 15)
		OXA-40 (*n* = 1)	None
		OXA-58 (*n* = 3)	(*n* = 3)
		OXA-72 (*n* = 4)	(*n* = 3)
		OXA-164 (*n* = 2)	None
*E. coli*	A (*n* = 2)	KPC-2 (*n* = 2)	(*n* = 2)
*K. pneumoniae*	A (*n* = 3)	KPC-3 (*n* = 3)	(*n* = 3)
*E. cloacae*	B (*n* = 2)	IMP-4, IMP-14 (*n* = 2)	(*n* = 2)
*S. marcescens*	B (*n* = 1)	IMP-13	(*n* = 1)
*E. cloacae*	B (*n* = 1)	NDM-1^a^ (*n* = 1)	(*n* = 1)
*E. coli*	B (*n* = 2)	NDM-1^a^, −3 (*n* = 2)	(*n* = 2)
*K. pneumoniae*	B (*n* = 1)	NDM-1^a^ (*n* = 1)	(*n* = 1)
*K. pneumoniae*	B (*n* = 2)	VIM-2 (*n* = 2)	(*n* = 2)
*K. oxytoca*	B (*n* = 1)	VIM-1, (*n* = 1)	(*n* = 1)
*E. cloacae*	B (*n* = 1)	VIM-4 (*n* = 1)	(*n* = 1)
*P. aeruginosa*	B (*n* = 2)	VIM-2 (*n* = 2)	(*n* = 2)
*K. pneumoniae*	D (*n* = 4)	OXA-48 (*n* = 4)	(*n* = 3)
*C. freundii*	D (*n* = 1)	OXA-162 (*n* = 1)	(*n* = 1)
*E. coli*	D (*n* = 1)	OXA-181 (*n* = 1)	(*n* = 1)
*K. pneumoniae*	D (*n* = 1)	OXA-204 (*n* = 1)	(*n* = 1)

^a^NDM-1/-6

### Hydrolysis assay directly from positive blood culture vials for the detection of carbapenemase-producing *Acinetobacter baumannii*, *Pseudomonas aeruginosa* and different *Enterobacteriaceae* isolates

To achieve faster results with the presented assay we set to run the hydrolysis assay on positive blood culture samples. Here, a total of 38 *A. baumannii* isolates were analyzed. Fourteen of the 38 isolates carried genes for a carabapenemase enzyme but did not show any activity in the MALDI-TOF MS hydrolysis assay (Table 1). Eight of the 29 OXA producing isolates (OXA-23, -40, -72, -164), five of the seven NDM-1 isolates and one VIM-2 *A. baumannii* isolate did not significantly show hydrolysis activity in the MALDI-TOF–MS (Table 1), although all the assays were performed at least in duplicate, revealing 63.2 % sensitivity.

Additionally, 25 multidrug-resistant *Enterobacteriaceae* and *P. aeruginosa* isolates spiked in blood culture vials were analyzed. Seven of 25 isolates produced a variant of OXA. The other enzyme variants are given in Table 1. All KPC-2/-3 producing isolates (*n* = 5) showed significant hydrolysis activity in the MALDI-TOF MS analysis. The hydrolysis activity of different class B enzymes was detected. Within the Ambler class D the OXA variants were detected in six different species, but one *E. coli* isolate with OXA-48 gene did not reveal significant hydrolysis activity in the MALDI-TOF MS hydrolysis analysis (Table 1). Taken together the present imipenem hydrolysis assay was able to detect carbapenemases in 24 of 25 (96 %) of blood cultures spiked with multidrug-resistant *Enterobacteriaceae* and *P. aeruginosa.* The 35 Gram-negative control isolates from positive blood culture vials showed no hydrolysis effects in the mass spectrometry.

## Discussion

The rapid detection of clinically important beta-lactamases (e.g. extended-spectrum beta-lactamases and carbapenemases) in routine diagnostic laboratories is crucial for initial antibiotic therapy for patients as well as for the prevention of the spread of beta-lactamase-producing bacteria in health care settings. MALDI-TOF mass spectrometry allows clinical microbiologists to discern unique protein signatures, abundantly ribosomal proteins, in order to identify the pathogens [16]. In addition, there is potential for the detection of resistance mechanisms [10]. The most common mechanism of resistance to beta-lactams is the hydrolysis of the amide bond of the beta-lactam ring [10]. This last application includes identification of the antibiotic itself and its modified/degraded derivatives [9, 12–14, 17]; detection of the resistance proteins within the cell [11, 18], and discovery of mutations within the resistance genes through mini-sequencing [19, 20]. Almost 4 years ago two studies on direct carbapenemase detection by MALDI-TOF MS hydrolysis assay were reported [9, 13]. An analysis of antibiotics and their degradation products that are smaller than 1000 Da by MALDI-TOF MS is possible using a specific sample preparation [21]. Several studies have assessed the utility of MALDI-TOF mass spectrometry for the identification of beta-lactams and beta-lactam degradation products within four hours, either from the culture media or positive blood culture vials [9, 12–15, 17, 22].

Jung et al. reported the detection of ampicillin and cefotaxime resistances in hydrolysis assays directly from blood cultures [22]. However, these assays can only detect the presence of beta-lactamases as a resistance mechanism. Detection of the actual proteins conferring resistance in such resistant bacteria by MALDI-TOF–MS seems to be more challenging since the protein signatures of bacteria are very complex. Camara and Hays [11] established a proof of principle for this methodology in which they dissected the differences between wild-type and ampicillin-resistant *E. coli* strains. After the cultivation of resistant strains in broth supplemented with ampicillin, proteins were extracted by using a formic acid-isopropyl alcohol-water solution and spotted onto a MALDI-TOF MS target by a sandwich method using sinapinic acid as a matrix. They identified a 29.000 Da peak in resistant strains confirming that this mass/ion peak represented a beta-lactamase.

Kempf et al. detected the carbapenemase-production of Gram-negative bacteria by performing the imipenem hydrolysis and Ultraflex MS analysis [23]. They were able to detect the peaks of imipenem (299 *m/z*) and the degradation product (255 *m/z*) in the mass spectra. Furthermore, Sparbier et al. [17] reported that in their imipenem hydrolysis assay they observed a complex of imipenem and the matrix (190.05 *m/z*) at 489 *m/z* was observed. In our assay, we detected the imipenem-matrix-complex at 479 *m/z* and the peak of the matrix could be demonstrated at 180 *m/z* (Fig. 1). In cases of carbapenemase-producing isolates the peak of this complex disappeared, but neither degradation products nor sodium variants of the antibiotic of imipenem were detectable, as confirmed by Sparbier et al. [17]. Moreover, we were not able to detect the instable hydrolysis product of imipenem.

From culture isolates our mass spectrometry-based imipenem hydrolysis assay correlated with the genotypic detection in 98 % of the cases, i.e., one OXA-48 *E. coli* isolate did not demonstrate the hydrolysis of imipenem. The hydrolysis activity of such enzyme might be weak as described previously [24]. The other OXA-48 producing bacteria belonged to *K. pneumoniae*; these showed excellent hydrolysis activity in our MALDI-TOF–MS based hydrolysis assay. For the positive blood culture isolates, we were able to detect the carbapenemase activity in only 24 (63.2 %) of total 38 *A. baumannii* isolates. For the remaining 14 isolates no interpretation was possible concerning their carbapenemase activity; these included the enzyme groups OXA (OXA-23, -40, and -164), NDM-1 and VIM-2. Thus, eight of the 29 OXA producing isolates, five of the seven NDM-1 producing isolates and one VIM-2 positive isolate were not correctly detectable. Even the repeated incubation and measurements did not reveal better interpretable results.

In our hydrolysis assay we relied solely upon the disappearance of imipenem + matrix peak to consider an isolate as carbapenemase-producer. Kempf et al. distinguished carbapenemase producing isolates with regard to disappearance of imipenem peak or an intact/hydrolyzed imipenem ratio of <0.5 [23]. Recent studies consider Liquid Chromatography-Tandem Mass Spectrometry the gold standard method for small-molecule detection, and its performance is well suited to the detection of small amounts of compounds present in highly complex matrices, such as serum or bacterial growth broths [25]. As also observed by Kulkarni et al. [25], the effects of ion suppression on carbapenem detection have not been reported for MALDI-TOF, and this could be particularly important when test interpretation relies upon the lack of detection of a compound. In several studies, the matrix peak has been used as an internal standard, but the chemical differences between MALDI matrix and carbapenems are substantial, and differential ionization effects may not be effectively determined without an appropriate internal standard.

## Abbreviations

MALDI-TOF MS: matrix-assisted laser desorption ionization time-of-flight mass spectrometry
MDR: multidrug resistant
KPC: *Klebsiella**pneumoniae* Carbapenemase
NDM: New-Delhi-Metallo-betalactamase
IMP: Imipenemase
GIM: German Imipenemase
OXA: Oxacillinase
